# The Sonoelastography and Functional Outcome of Upper Extremity after Kinesiotaping on the Spastic Forearm in Patients with Subacute Stroke

**DOI:** 10.1155/2023/1730491

**Published:** 2023-01-16

**Authors:** Shih-Jung Lai, Yu-Chi Huang, Po-Cheng Chen, Jia-Ying Wu, Chau-Peng Leong

**Affiliations:** Department of Physical Medicine and Rehabilitation, Kaohsiung Chang Gung Memorial Hospital and Chang Gung University College of Medicine, Kaohsiung, Taiwan

## Abstract

**Objectives:**

This study is aimed at exploring the feasibility of sonoelastography on muscle stiffness of spastic forearm and evaluating the improvement of functional performance in patients with poststroke spasticity (PSS) after receiving kinesiotaping (KT) and rehabilitation.

**Methods:**

According to the spastic levels (using modified Ashworth scale (MAS)) of the affected upper extremity, 59 patients with stroke were allocated into two groups, group A (MAS 0–1): 31 patients (14 men and 17 women; mean age: 60 years) and group B (MAS 1+–2): 28 patients (22 men and 6 women; mean age: 51 years). The Brunnstrom motor recovery stage at the wrist/distal parts in groups A and B was stage 3/3.5 and stage 2.75/3. We evaluated the Brunnstrom stage, spastic levels by MAS and modified Tardieu scale (MTS), and Fugl-Meyer Assessment for upper extremity (FMA-UE). We also evaluated the muscle spasticity of flexor carpi radialis (FCR), flexor carpi ulnaris (FCU), and flexor digitorum superficialis (FDS) muscles using sonoelastography with shear wave velocity (SWV). We applied KT for 20 patients in group B, comparing the changes in sonoelastography and functional outcomes between KT and without KT interventions.

**Results:**

Both the MAS and MTS scales were moderately correlated with the SWV in forearm muscles on hemiplegic side (*r* = 0.336–0.554) After KT intervention, the SWV in FCR decreased (*p* = 0.028). Muscle spasticity was reduced (*p* < 0.01), and distal part of the Brunnstrom stage and FMA-UE were increased (*p* = 0.045 and *p* = 0.001). In patients without KT intervention, only the MTS degree reduced (*p* = 0.026).

**Conclusions:**

The SWV of sonoelastography could objectively assess the reduction of muscle stiffness of the affected forearms in patients with PSS after KT intervention. *Advances in Knowledge*. Sonoelastography could be a quantitative method to follow up for therapeutic effect of the spastic forearm.

## 1. Introduction

Poststroke spasticity (PSS) is a common complication in patients with stroke with brain injury, which may cause impaired cocontraction between agonists and antagonists, weak limbs, and impaired coordination [1]. Previous studies found that the prevalence of PSS varied from 25% to 42.6% in patients with stroke who developed spasticity according to their different disease durations ranging from 6 weeks to 6 months after a brain insult [2, 3]. Spasticity was associated with lower Barthel Index scores with impaired activities of daily living, stroke-related pain, severe limb paresis, and sensory loss [2]. Patients with stroke with PSS might suffer from a decline in their ability to perform activities of daily living and quality of life. In the socioeconomic burden of PSS, a 4-fold increase in health care costs was reported in patients with stroke with PSS compared to patients without spasticity [4]. Therefore, reducing the increased muscle tone with effective spasticity intervention and sensory facilitation during rehabilitation could decrease the level of spasticity and disability in stroke survivors, which might improve their functioning and quality of life and might diminish the burden on their caregivers [4].

The modified Ashworth scale (MAS) and modified Tardieu scale (MTS) have been widely used in patients with stroke with PSS. The MAS scale is easier for the examiner to measure; however, it is more difficult to quantify the score. The interrater and intrarater reliability of MAS has been calculated by several researchers with incompatible results for different muscle groups [5]. For evaluating PSS of the upper extremity, Ansari et al. mentioned that the MAS had poor interrater reliability for evaluating elbow flexors in patients with stroke with hemiplegia [6]. Li et al. mentioned that the MAS demonstrates moderate to substantial test-retest reliability and interrater reliability in measuring spasticity [5]. The MTS has been considered a better alternative than the MAS for assessing spasticity. Singh et al. demonstrated that the intrarater reliability of the MTS was good in a large sample of patients with stroke [7]. Sonoelastography is a developing imaging technique that can measure tissue elasticity and be more frequently applied to muscle stiffness in patients with brain insults [8]. Some researchers have recently used sonoelastography and shear wave velocity to assess muscle stiffness in patients with PSS over the upper limbs; however, most studies have been performed on the biceps brachii muscle [8, 9]. Liu et al. evaluated biceps brachii muscles [9], and Yasar et al. evaluated wrist and finger flexors in patients with stroke via sonoelastography [8]. They all found that muscle stiffness increased on the hemiplegic side in patients with stroke compared to the normal side. Therefore, they considered that sonoelastography could provide an objective evaluation of muscle spasticity on the affected side in patients with stroke.

For spastic forearms, interdisciplinary complex rehabilitation interventions are the mainstay to treat functional deficits after acute stroke [10]. Stretching and range of motion exercises can increase muscle length and maintain joint motion. Neuromuscular or transcutaneous electrical stimulation has also been shown to positively affect spasticity management [11]. Kinesiotaping (KT) has been implemented as an adjuvant therapeutic technique for facilitating motor performance and reducing PSS in hemiplegic patients. In a previous study, KT increased proprioception by enhancing the stimulation of cutaneous mechanoreceptors [11–13]. KT has been proposed to enrich sensory inputs, decrease spasticity by proprioception feedback, and relieve abnormal muscle tension [13]. In addition to rehabilitation, KT can provide more positive effects both on PSS and on functional motor performance of the affected hand in patients with subacute stroke [11, 12, 14].

In this study, we aimed to explore the feasibility of sonoelastography on muscle stiffness of spastic forearm muscles by quantitative assessments and to investigate the role of sonoelastography in assessing the changes in the wrist and finger muscle stiffness and the improvement of functional performance in patients with subacute stroke with PSS after receiving combined kinesiotaping and rehabilitation. We wished to combine KT with a rehabilitation program to promote functional outcomes in patients with PSS. To our knowledge, this is the first study to assess objective changes in muscle stiffness before and after interventions in patients with stroke with spastic forearm using sonoelastography.

## 2. Materials and Methods

A retrospective study was conducted at the rehabilitation unit of a tertiary medical center in Taiwan. All procedures in this study were reviewed by the institutional review board of Chang Gung Memorial Hospital. Each participant agreed with the consent form before participating in the study. We enrolled 59 hemiplegic patients with stroke (36 men and 23 women) in this study. Each patient with stroke was diagnosed based on the history, physical examination, and brain imaging evaluation. The inclusion criteria were as follows: (1) the patients had a stroke with hemiplegia in the affected upper extremities (duration since stroke ranged from 2 to 12 months), (2) patients who could perform hand grasping and could slightly perform finger extension and minimal wrist extension, and (3) free passive range of elbow and wrist motions without contracture or deformity of the affected upper limbs. The exclusion criteria were age less than 18 years or more than 80 years, had a history of upper extremity tendon/neuromuscular injury or any systemic neuromuscular disorder, and communication difficulty related to cognition or language impairment.

Physical findings and sonoelastography of the affected upper extremities were assessed and recorded. According to the spastic levels of the affected upper extremity, the patients were allocated into two groups. In group A, patients had normal to slight spasticity (MAS 0–1) on the hemiplegic upper limb after stroke. Patients with increased spasticity (MAS 1+–2) in the affected upper limb were included in group B. We collected all clinical parameters, including age, sex, hemiplegic side, and duration since the stroke. In addition, Brunnstrom motor recovery stage, spastic levels of the upper extremity involving the wrist and hand joints by MAS and MTS, and the Fugl-Meyer Assessment for upper extremity (FMA-UE) for each participant were also evaluated.

Brunnstrom motor recovery stage [15] is defined as follows: stage I, flaccid with no voluntary movement; stage II, spasticity begins to develop with the presence of minimal voluntary movement; stage III, voluntary control of movement within a flexor synergy pattern and moderate to severe spasticity; stage IV, spasticity begins to decline with more selective activation of muscles outside the flexor synergy; stage V, mastery of more selective and independent muscle activation without dominance by flexor synergy; stage VI, individual joint movement and well-coordinated movement are present without abnormal muscle tone. The five-point MAS [5, 16] was used for measuring spasticity of the elbow flexors and wrist flexors, and it is ranked as follows: 0, no increase in muscle tone; 1, a slight increase in muscle tone, manifested by a catch at the end of range of motion (ROM); 2, marked increase in muscle tone through most ROM such that the affected limb is easily movable; 3, a considerable increase in muscle tone but difficulty in passive movement of the affected limb; and 4, rigid affected limb. In the MTS level [5, 17], a score of 0 represents no resistance in the muscle belly while being stretched; 1, a slight resistance during stretching without a catch; 2, an obvious catch at a specific angle while being stretched, followed by a release until the end range; 3, a short period of clonus while stretching; 4, a longer clonus for more than ten seconds; 5, joint contracture or extreme difficulty in moving the joint. The MTS degree represented the stretch reflex, dependent on velocity. The difference in wrist angles between slow passive movement and fast speed of catch and release was measured [8, 18, 19]. In the FMA-UE [20], part A included the upper extremity, indicating proximal motor function. Parts B and C included wrist and hand motions, representing the distal hand function. Total scores ranged from 0 to 54.

The clinical characteristics of the patients with stroke with hemiplegia are presented in Table 1. There were 31 patients with stroke without significant spasticity (MAS 0–1) in group A (14 men and 17 women; mean age: 60 years) and 28 patients with stroke with significant spasticity (MAS 1+–2) in group B (22 men and 6 women; mean age: 51 years). Sixteen patients with left hemiplegia and 15 with right hemiplegia were found in group A, and 10 patients with left hemiplegia and 18 patients with right hemiplegia were in group B. The median duration from stroke onset in groups A and B was 125 and 163.5 days, respectively. The Brunnstrom motor recovery stage at the wrist/distal parts in groups A and B was stage 3/3.5 and stage 2.75/3. There were significant differences in age, sex, and duration since stroke onset between the two groups (*p* < 0.05).

We also evaluated the muscle spasticity of the flexor carpi radialis (FCR), flexor carpi ulnaris (FCU), and flexor digitorum superficialis (FDS) muscles at both forearms using sonoelastography with shear wave velocity (SWV). Musculoskeletal sonography (Acuson S2000, Siemens, USA with a 9L4 transducer) examinations were performed by one physician with more than 10 years of experience. The participant needed to follow the instructions for neutral positioning of their forearm with elbow flexion at 90 degrees while sitting upright. The affected upper arm was placed with the elbow placed at 90-degree flexion. The wrist was placed in a neutral position. To maintain these required positions at both the elbow and wrist, positioning splints for elbow 90-degree flexion and a cock-up splint for maintaining the wrist in a neutral position had to be applied on the affected upper limb during sonoelastography. In SWV, a 4–9 MHz linear transducer was used during the examination. To standardize the location of the sonographic assessment, the probe was placed on the upper one-third of the forearm for the FCU, FCR, and FDS muscles. The region of interest was set in the central part of the muscle bellies, while the transducer was placed along the longitudinal direction of the muscle fibers. Seven measurements were recorded, and the median number of measurements was used for further analysis. We then applied kinesiotaping for 20 patients in group B who suffered from hemiplegia and significant spasticity, comparing the changes in sonoelastography and functional outcomes between KT and without KT interventions in group B after 3 weeks of KT combined with rehabilitation.

## 3. Data Analysis

Excel (Microsoft Corporation, Albuquerque, New Mexico, USA) and IBM SPSS 20 (IBM, Armonk, NY, USA) were used for the statistical analyses. Pearson's correlation was used to examine the relationship between MAS, MTS, and SWV. The chi-squared test was used to analyze the categorical variables, including sex and hemiplegic side. The Mann–Whitney *U* test was used to compare between-group differences in numerical variables, including age, duration since the stroke, Brunnstrom stage in the wrist and distal part, and FMA-UE. The Wilcoxon signed-rank test was used for within-group comparisons of SWV, MAS, MTS degree and level, Brunnstrom stage, and total score of FMA-UE. All results are presented as the median number (±interquartile range). Statistical significance was set at an alpha level of *p* < 0.05.

## 4. Results

The correlations of SWV with MAS and MTS degree in hemiplegic patients with stroke before intervention are shown in Table 2. In Table 2, the MAS score was moderately correlated with SWV measured in the FCR, FDS, and FCU on the hemiplegic side of patients with stroke (*r* = 0.395, 0.336, and 0.380, respectively). Furthermore, the MTS scale also had a moderate correlation with SWV measured in FCR, FDS, and FCU (*r* = 0.554, 0.469, and 0.417, respectively).

In group A (Table 3), the SWV of the FCR, FDS, and FCU muscles on the normal forearm were 2.46, 2.32, and 2.41, respectively. Those on the affected side were 2.45, 2.16, and 2.21, respectively. No significant differences were found between the normal and affected forearms in group A. In group B, the SWV of the FCR, FDS, and FCU muscles on the normal forearm were 2.58, 2.18, and 2.45 m/s. The velocities on the hemiplegic side were 2.87, 2.44, and 2.56 m/s. A significantly higher speed was found in the SWV of the FCR and FDS muscles on the hemiplegic side than on the normal side (*p* < 0.01).

We compared the changes in sonoelastography and functional outcomes between KT and without KT interventions in 28 patients with stroke with hemiplegia and significant spasticity (Table 4). After KT intervention, the SWV in the FCR significantly decreased. Muscle spasticity, including MAS and MTS degree/level, was reduced (*p* < 0.01), and the distal part of Brunnstrom motor recovery stage and FMA-UE were significantly increased (*p* = 0.045 and *p* = 0.001, respectively). In patients without KT intervention, no significant differences were found in the SWV of all muscles on sonoelastography and functional outcomes, except for the MTS degree (*p* = 0.026).

## 5. Discussion

Upper extremity spasticity after stroke impeded motor recovery, especially wrist and hand function, during rehabilitation. In this study, we used the shear wave velocity of sonoelastography to objectively assess muscle stiffness in affected forearm muscles in patients with PSS during rehabilitation combined with kinesiotaping intervention. We found significantly increased SWV in both the flexor carpi radialis and flexor digitorum superficialis muscles on the hemiplegic forearm in patients with stroke with significant spasticity, but no significant finding on flexor carpi ulnaris muscle. Significantly decreased MAS and MTS of the forearm and SWV in the FCR muscle were found after KT. In addition, both Brunnstrom motor recovery stage in the distal part of the affected upper limb and FMA-UE score were improved in patients with spasticity after the intervention.

In previous studies investigating muscle spasticity after stroke, some researchers found that SWV at the biceps muscle was associated with passive range of motion on the affected upper extremity [21, 22]. Higher SWV was also related to increased muscle tone and poor function in the affected upper limb after stroke. In the spastic forearm, Yasar et al. used the elasticity index and ratio to evaluate the spasticity of the wrist and finger flexors and revealed an increase in these parameters compared to the normal side [8]. All these findings supported that sonoelastography could be an objective tool to assess PSS, and it might be a practical method to measure the changes in muscle spasticity clinically. In our study, we applied SWV of sonoelastography and had similar results and found increased SWV of the FCR and FDS muscles on the affected forearm in patients with stroke with PSS. We reinforced that SWV on sonoelastography could also provide a convenient and quantitative method to measure the muscle stiffness of forearm muscles in patients for evaluation of PSS.

KT is an adjuvant and beneficial therapy for managing PSS of the upper extremity during stroke rehabilitation. It could improve upper extremity motor function in patients with stroke with hemiplegia. In previous reports, kinesiotaping was shown to significantly reduce PSS and enhance upper extremity performance in hemiplegic patients with PSS [12]. After 3 weeks of KT intervention, we found a significant decrease in muscle spasticity clinically by MAS and MTS evaluations in this study. Furthermore, sonoelastography revealed a significant reduction in SWV in the FCR muscle of the affected forearm after KT. Since PSS improved after combined KT and rehabilitation, the patients had improved motor performance on both the Brunnstrom stage at the distal part of the affected forearm and the FMA-UE score.

This study is the first to explore the role of SWV in improving PSS in the affected forearm after KT application in patients with stroke with hemiplegia during rehabilitation. Therefore, SWV of FCR might provide an objective and alternative method to observe the change in muscle spasticity in patients with stroke with spastic forearm after spasticity intervention.

The limitations of this study are as follows. All patients were recruited from a single medical center. Only 20 patients with mild to moderate spastic forearm received KT to reduce PSS; hence, it was a small sample size and could not present population-based patients with stroke with different severities of spasticity. Due to the small number of patients, the neuromotor reeducation treatment was not described, and it did not take into account all the variables that may be caused by the stroke with the associated consequences. The follow-up research should be with evaluations in the first 60 days and repeat the final evaluation after one year after the onset of the acute event. This could be a good baseline for other studies.

In the future, we hope to investigate patients with stroke with more severe spasticity and to explore the clinical applications and changes in SWV on sonoelastography to evaluate the spastic limbs before and after treatment.

## 6. Conclusion

In summary, significantly increased SWV at both the wrist and finger flexor muscles was found on the hemiplegic forearm in patients with stroke with mild to moderate spasticity. The SWV of sonoelastography could objectively assess the reduction of muscle stiffness in the FCR muscles of the affected forearms in patients with PSS after rehabilitation and KT. After effective reduction of muscle spasticity, better motor recovery and performance of the upper extremities were obtained in hemiplegic patients with stroke. We believe that sonoelastography could be an objective and quantitative method to follow up on the therapeutic effect of different interventions for the spastic forearm.

## Figures and Tables

**Table 1:** Clinical characteristics of patients with stroke with hemiplegia.

	Group A (*n* = 31)	Group B (*n* = 28)	*p*
Age, years (median (IQR))	60 (12)	51 (16.5)	0.004^∗^
Sex, male/female, *n*	14, 17	22, 6	0.006^∗^
Hemiplegic side, *n* (%)			
Left	16 (51.6)	10 (35.71)	0.176
Right	15 (48.4)	18 (64.29)	
Duration since stroke, days (median (IQR))	125 (63)	163.5 (91.25)	0.042^∗^
Brunnstrom stage (median (IQR))			
Wrist	3 (2.5)	2.75 (1)	0.284
Distal	3.5 (3)	3 (0.88)	0.207
FMA-UE (median (IQR))	19 (27)	14 (11.5)	0.085

Chi-squared test was used for sex and hemiplegic side. Mann–Whitney *U* test was used for between-group comparisons of age, duration since the stroke, Brunnstrom stage in the wrist and distal part, MTS degree and level, and FMA-UE. IQR: interquartile range; FMA-UE total: total score of part A, part B, and part C in Fugl-Meyer Assessment for upper extremity. ^∗^*p* < 0.05.

**Table 2:** Correlations of SWV with MAS and MTS degree in hemiplegic patients with stroke before intervention.

	MAS	MTS degree
*SWV*		
FCR (median (IQR))	0.395	0.554
FDS (median (IQR))	0.336	0.469
FCU (median (IQR))	0.380	0.417

SWV: shear wave velocity; MAS: modified Ashworth scale; MTS: modified Tardieu scale; FCR: flexor carpi radialis; IQR: interquartile range; FDS: flexor digitorum superficialis; FCU: flexor carpi ulnaris.

**Table 3:** Comparisons of sonoelastography between normal and hemiplegic sides in patients with stroke with hemiplegia before intervention.

	Group A (MAS 0–1; *n* = 31)			Group B (MAS 1.5–2, *n* = 28)		
	Normal side	Hemiplegic side	*p*	Normal side	Hemiplegic side	*p*
*SWV*						
FCR (median (IQR))	2.46 (0.43)	2.45 (0.67)	0.563	2.58 (0.47)	2.87 (0.485)	0.003^∗^
FDS (median (IQR))	2.32 (0.34)	2.16 (0.56)	0.161	2.18 (0.39)	2.44 (0.465)	0.009^∗^
FCU (median (IQR))	2.41 (0.51)	2.21 (0.61)	0.158	2.45 (0.43)	2.56 (0.443)	0.336

Wilcoxon signed-rank test was used for comparisons of SWV. MAS: modified Ashworth scale; SWV: shear wave velocity; FCR: flexor carpi radialis; IQR: interquartile range; FDS: flexor digitorum superficialis; FCU: flexor carpi ulnaris.

**Table 4:** Comparisons of sonoelastography and functional outcomes after KT and without KT interventions in patients with stroke with PSS.

	With KT (*n* = 20)			Without KT (*n* = 8)		
	Baseline	3^rd^ week	*p*	Baseline	3^rd^ week	*p*
SWV						
FCR (median (IQR))	2.87 (0.5)	2.775 (0.75)	0.028^∗^	2.87 (0.8)	2.66 (0.41)	0.484
FDS (median (IQR))	2.42 (0.47)	2.275 (0.52)	0.126	2.49 (0.57)	2.51 (0.21)	1.000
FCU (median (IQR))	2.59 (0.66)	2.665 (0.7)	0.751	2.52 (0.31)	2.31 (0.55)	0.161
Spasticity						
MAS	2 (0.5)	1.5 (1)	0.004^∗^	1.75 (0.5)	1.5 (0.75)	0.109
MTS degree	65 (47.5)	47.5(30)	0.008^∗^	55 (40)	45 (28.75)	0.026^∗^
MTS level	2 (0.38)	1.5 (1)	0.009^∗^	2 (0.75)	1.75 (1)	0.102
Brunnstrom stage (median (IQR))						
Wrist	2.75 (1.38)	3 (1.5)	0.305	2.75 (1.75)	2.5 (2.5)	0.655
Distal part	3 (0.88)	3.5 (1.38)	0.045^∗^	3 (1.63)	3.25 (1)	0.276
FMA-UE (median (IQR))	14 (12.5)	20.5 (15.5)	0.001^∗∗^	8.5 (8.75)	9 (7.5)	0.785

Wilcoxon signed-rank test was used for within-group comparison of ARFI, MAS, MTS degree and level, Brunnstrom stage, and total score of FMA-UE. *p*: within-group comparisons of the results between two assessing times. KT: kinesiotaping; PSS: poststroke spasticity; SWV: shear wave velocity; FCR: flexor carpi radialis; IQR: interquartile range; FDS: flexor digitorum superficialis; FCU: flexor carpi ulnaris; MAS: modified Ashworth scale; MTS: modified Tardieu scale; FMA-UE total: total score of part A, part B, and part C in Fugl-Meyer Assessment for upper extremity; ARFI: acoustic radiation force impulse. ^∗^*p* < 0.05.

## Data Availability

The data used to support the findings of this study are available from the corresponding author upon request.
